# Ante-Natal Physiology

**Published:** 1911-03-15

**Authors:** N. S. Hoff


### ANTE-NATAL PHYSIOLOGY.

BY N. S. HOFF, D.D.S.

---

Our Anatomic knowledge of prenatal conditions of a developing foetus in its various stages is reasonably definite, but the physiologic processes, especially when they result in pathologic or abnormal conditions, are not so readily demonstrated. In fact we are largely compelled to resort to logical deductions from known adult functions and consequently to hypothetical conclusions. No scientific work with reference to the teeth has made clear the occasional serious malformations of the teeth and jaws, and very little that is conclusive accounts for the ordinary defects of tooth calcification. We can only hope therefore to call attention to the probable and important incidents in ante-natal existence, with the hope that future research may more clearly establish the essential facts.

**Foetal Development.** The Foetal period of life is chiefly one one of development dependent on maternal sustenance and to that extent prejudiced. The organs and tissues of the foetus have largely the function of distributing and assimilating the predigested pap from the maternal blood supply. It is therefore easy for us to believe that accidental interruptions should have much to do with abnormal developmental results, and to consider it remarkable that so many children are normally developed at birth, when we recall the innumerable accidents to which pregnant women are liable. We must also take into consideration the facts that prejudice the normal beginning of foetal life itself. If the established principles of heredity are to be reckoned into the problem we should not only go back several ancestral generations, as we have frequently seen cases cited in our literature of freak tooth anomalies appearing in a long line of ancestors. We shall not at this time undertake to discuss this subject as it can have little bearing on the object of this study. It will be enough for us to consider the influence of the more immediate foetal environment. For our purpose it will be sufficient to state that it is generally conceded that actual disease may not be transmitted by the sperm and ovum but that impaired tissue results during the embryonic and developmental periods. The embryonic period begins with the impregnation of the ovum and lasts for six weeks, during which time tissue differentiation takes place and the important foetal organs are developed. The balance of the intra-uterine period is devoted to the growth of the foetus and the complete development of the vital organs necessary for independent or individual existence. During the embryonic period there are no dental organs of consequence developed and it would be impracticable to even suggest hygienic treatment of a prophylactic character. The beginnings of dental development are concurrent with foetal or organized function, and we find the first indication of future tooth organs in the so-called dental ridge which appears about the sixth week of pregnancy. As this ridge is developed from two different classes of germ cells, the epiblastic and the mesoblastic layers of the embryo, and as the two kinds of cells enter into such a harmonious combination to produce the entire tooth with its several widely differing structures, we can readily conceive that there can be many opportunities in the developmental process for accidents which should result in abnormal or faulty development. It is marvelous to find so nearly normal results when we think of the great possibilities for interrupted cooperation. Should a pathologic influence be present either of the transmitted type, or of a temporary maternal affection, it would seem that no typically normal development could be expected. It is the knowledge of these pending possibilities that prompt us to suggest that prophylactic hygiene of a more rigid character than is customary should be promulgated in behalf of good teeth.

**Ante-Natal Hygiene.** Preventive medicine has for many years been a mere word for medicine writers to juggle; but today we find current medical literature well supplied with reports of well directed efforts to enforce sanitary principles in private practice and in a larger manner through innumerable philanthropic and civic agencies. Textbooks, chairs of instruction in the better medical schools, and scientific researches in specially endowed institutions are advocating the prevention as well as the cure of disease. So far for the most part, these endeavors are directed to the saving of children from diseases which cause the death of nearly one half of all children born during the first two years of their lives. The cause of this great infant mortality is worth knowing as it has become a serious loss to the world, and in some of the European countries efforts are being made to take better care of pregnant women, that their children may be normally developed in foetal life and that they may pass through the trying ordeal of infant existence free from hazardous diseases. Ante-natal hygiene or prophylaxis is bound to be the logical outcome of this new research and no specialty of the healing art will be more benefitted by an improvement in the case of pregnant women than dentistry as it will surely result in better physically developed dental organs.

About all the advice modern textbooks on obstetrics give to pregnant women, is, that they should more carefully observe all the general rules that make for good health. This advise of course is good but it certainly is not sufficiently specific to render it effective or capable of rigid enforcement. It is a critical time for both mother and child, as we know that dentally the mother often suffers irreparably during such periods, and when the developing teeth overdraw the natural food supply to that extent they also must be undersupplied.

We are confident that a well directed regime of prophylactic hygiene and food supply would so fortify the tooth-forming organs as to produce the most desirable local and systemic results for both mother and child.

**Diet in Pregnancy.** We have only to refer to the great difficulty in deciding what shall be the proper diet for the various conditions in adult life, to realize that the task of specifying an ideal diet for pregnant women is even more hopeless. The old adage that "the pregnant women should eat for two" has been the cause of much harm in the development of monstrosities to say nothing of bringing great injury to the mother in childbirth. The growing foetus of course would at no time need the same kind or proportion of food as the mother. Another popular notion has been that thefoetus grows at the expense of the mother's structural form and therefore the mother's teeth must give up their calcific elements to supply this lack, if need be, in the mother's food. Therefore the cause for increased caries of the mother's teeth during pregnancy is due to lack of lime salt in her food. This is a popular conception that has no scientific basis upon which to rest; it is only an easy way of shifting the responsibility for a rational prophylactic regimen. In general and simple terms, it would be much better to state the matter tersely, that the mother should eat such food as her normal appetite craves and in such amounts and at such periods as shall preserve an evenly balanced equilibrium of health. In Hearst's Text Book on Obstetrics, (1900 page 189,) is recorded the case of a woman who took two quarts of milk daily in addition to her regular meals, and her confinement was attended with great labor and the child weighed eleven and three-quarters pounds. Other cases are on record where lime salts in chemical solutions are taken continuously and natural labor was made difficult.

D. Noel Paton made a research to determine with what accuracy the result of feeding could be predetermined from a dietary regulation. In well fed guinea pigs he found that each gram of mother's weight produced from 0.35 to 0.40 grams of offspring. Other animals of same conditions less bountifully fed never produce more than 0.22 grams of offspring, from which it would appear that actual weight may be influenced in uterine development by increasing the mother's food supply and so long as it does not exceed the normal requirements of good health it may be safely done. The opinion of many writers, however, seems to be that new born babies of average weight have greater vitality and are more likely to live and develop normally than those of overweight.

The "longings" of pregnant women for certain articles of food is the expression of a true physiological need. It is usually expressed in a craving for some peculiar salt or flavor, an acid or alkaly, that can be had only through some appropriate food; as in pickles, sauer kraut, smoked fish, condiments etc., which indicates incompleteness in the ordinary diet of the family. It should not however be inferred that a capricious diet should be sanctioned to the extent that it would produce disturbance of digestion or tend to become habitual. In test cases refusal to comply with these natural desires have been followed by no detrimental consequences while generally an excessive catering to them often results disastrously. A plain and wholesome diet that suits the individual and that but slightly emphasizes some particular need of a former experience is more to be commended than to risk securing some special objection by abnormal feeding.

**Influence of the Mothers Diet on the Offspring.** While much has been written on this subject in general terms most obstetricians confine their statements to general rather than specific directions as to diet. Obviously this should be so, since the innumerable variations in domestic life and physical conditions preclude anything like a mathematical statement that all could follow. Some scientific work, however, has been done that makes it possible to classify and adapt to some extent specific measures. Prochownick, in *Therapeutische Monatshefte* Vol. xv, 1901, Nos. 8 and 9, shows that the size, weight and osseous development of the foetus can be so influenced in cases of pelvic contraction of from three to four inches, as to make full term pregnancy practicable where premature labor had to be induced in previous accouchements. His proofs rest on a series of forty-eight persons and sixty-two confinements. Briefly stated his tests were based on a set diet, such as follows: The patients were given for breakfast, a small cup of coffee, 25 grams of bread and about an ounce of butter. For dinner, some kind of meat or an egg, or fish with suitable sauce, green vegetables, salad, cheese. For supper about the same in substance as dinner with bread and butter at pleasure. No soups, water, potatoes, puddings, sugar or beer were allowed. 300 to 400 c. c. of Moselle or red wine might be partaken during the day, but the total fluid should not exceed 500 c. c. per day. Slight variations for individual requirements were permitted. The author claims that by such a dietary the usual increase in bulk of the foetus was restrained and the ossification of the cranial bones was delayed so that they would permit the required compression in passing through the abnormally restricted pelvic outlet. It has also been shown by Schkarin, in "*Monatschrift fuer Kinderheilkunde*" Vol. ix, 1910 p. 65, that when the mother's diet is faulty the fœtus seems to have the faculty of obtaining the pabulum necessary for its own growth at the expense of the mother's nourishment; and he also found on analizing the mother's milk that there was but slight variations in its composition on changes of diet, except after long continued feeding. His experiments were made on rabbits. A group of nursing rabbits were fed on their ordinary vegetable diet, another group on milk and eggs, and a third group on milk and meat. The weight of the young was accurately estimated at stated intervals, and later the ashes were weighed and the phosphorus content determined. The result showed but slight difference from the different methods of feeding. This evidence would seem to justify the common practice among the ignorant classes of giving little or no thought to the quality of their food; but Schkarin found that where a proper diet was ignored the mother's health first suffered and the child later on was also markedly influenced. The points which these two researches seem to make for good and poor teeth are: first, that calcification of the teeth may be influenced by attention to the diet of pregnant mothers.

Whether it can be abnormally exaggerated or not, as development in the bony system has been shown possible, we have no conclusive proof to offer; but from the fact that foetal development may be retarded and controlled without producing maldevelopment, under a strict dietary regimen, we are certainly justified to conclude that a dietary including tooth elements must make for a better organized and calcified tooth. The second point is this: many of the dental aberrations which we encounter are probably due to the fact that during the foetal and nursing periods the mother has been, not so much underfed, but poorly fed. In other words demands were made on the mother by the child which she could not supply and in time the defect showed its self in abnormal dentition. "Physiologists tell us that the epithelial cells of the mammary glands collect selectively all the inorganic materials from the blood in exactly the proportions required by the offspring that it may grow naturally to the type of its parents, therefore the proportions assimilated by the child will be practically the same as is found in the mothers milk."—*Shaw and La Fetra* p. 365. This statement it seems ought to make it very plain that in order to secure proper dentition it will be necessary to exercise some care in preparing the mother for the important function of giving her offspring a chance to develop normally as well as typically.

**The Time for Intervention.** The first and most important fact to be established in preventive dentistry is the possibility of normal and typical dentition. There can be no question as to the possibility of practically a perfect dentition, but it is so rare in practice as to raise the query of the practicability of securing it by any kind of hygienic treatment unless this can be accomplished it would seem that little hope can be held out for better health through present therapeutic resources only. The old notion that it takes at least two generations to change one's individuality is fast giving way to the more intelligent conception that the purpose is the main requirement. Primarily it seems logical for us to begin our work with the immediate parents and during the generative periods. We must first see to it that there is a healthy and competent tooth germ capable of producing a perfectly calcified tooth. For this we must begin with intra-uterine life and provide the mother through hygienic surroundings and proper diet with the nourishing elements with which to accomplish this purpose. This does not mean a diet shall be provided that is so refined that a depressing mental effect is made upon the mother, but she should be seriously impressed with the responsibility which this opportunity offers her of bestowing upon her offspring a most valuable heritage and her willing cooperation should in this way insure a successful dental development. Whatever the particular schedule of diet may be, it should contain well balanced proportions of the three great food subdivisions; nitrogens, carbohydrates and fats. For illustration, an ideal diet should contain large proportions of vegetables and fruits, especially when seasonable. These foods will supply many of the inorganic salts required is such form as to make them readily assimilable by the growing foetus, and it will be found that the usual abnormal cravings of the mother for certain food qualities will be absent unless the mother's own system is lacking in these elements. There is a prevailing notion with hygienists that too much starch is supplied to pregnant women at the expense of too little fruit and vegetables. The habitual use of canned fruit should be substituted with fresh vegetables.

**Tooth Foods.** In order that we may get some idea of what the food needed for producing enamel may be so that it in particular may be provided in our food supply we take the following table from Marshall's textbook on *Operative Dentistry* (p. 38.)

CHEMICAL COMPOSITION OF ENAMEL—BY VON BIBRA.

| | In Man | In Woman |
|---|---|---|
| Calcium Phosphate and Fluoride | 89.82 | 81.63 |
| Calcium Carbonate | 4.37 | 8.88 |
| Magnesium Phosphate | 1.34 | 2.55 |

| | In Man | In Woman |
|---|---|---|
| Other Salts | .88 | .97 |
| Cartilage | 3.39 | 5.97 |
| Fat | .20 | trace |
| | 100 | 100 |
| Total Inorganic Material | 96.41 | 94.03 |
| Total Organic Material | 3.59 | 5.97 |

Shaw and La Fetra, p. 365, report the results of an analysis by Soldner on the proportions of inorganic salts found in mother's milk ashes as:

$CaO = 13.9$, $K_2O = 32.4$, $MgO, = 1.9$, $P_2O_5 = 11.4$, $Cl = 27.7$.

The following will indicate some sources for the desirable elements to be had from the ordinary foods:

Albumen, from cheese, roast veal, ham, sausage, fish, beef, peas; fats from butter, bacon, sausage, pork, yolk of egg, cream cheese. Carbohydrates, from zweiback, biscuit, chocolate, dried fruit, peas. Salts, from salted fish, smoked ham, sausage, cream cheese, cocoa. Cellulose, from dried and green fruits, peas, salads.

